# Cholinergic inhibition disrupts retrieval-specific phosphorylation of CaMKII-α and CaMKII-β alongside ERK hyperactivation in the hippocampus during novel object recognition memory

**DOI:** 10.1186/s12868-026-01027-0

**Published:** 2026-07-20

**Authors:** Majid Reza Farrokhi, Alireza Askari, Roksana SoukhakLari, Maryam Moosavi

**Affiliations:** 1 Shiraz Neuroscience Research Centre, Shiraz University of Medical sciences, Shiraz, Iran; 2 Students Research Committee, School of Medicine, Shiraz University of Medical Sciences, Shiraz, Iran; 3 Department of Cardiology, Medical University of Graz, Graz, Austria

**Keywords:** Recognition memory retrieval, Cholinergic inhibition, CaMKII-α, CaMKII-β, ERK, Novel object recognition, Hippocampus

## Abstract

**Background:**

Alzheimer’s disease (AD), the most common form of dementia, is characterized by cholinergic dysfunction and early impairments in episodic and recognition memory. Although retrieval failure represents a hallmark of cognitive decline in AD, the task- and phase-specific molecular mechanisms underlying cholinergic-dependent memory retrieval remain poorly defined.

**Objective:**

This study investigated whether acute muscarinic cholinergic blockade disrupts the retrieval phase of recognition memory. We also examined the associated regulation of hippocampal calcium/calmodulin-dependent protein kinase II (CaMKII) isoforms α and β, together with extracellular signal-regulated kinase (ERK) signaling.

**Methods:**

Adult male SWR/J mice were trained in the NOR paradigm and administered scopolamine (1 mg/kg, i.p.) or saline 30 min prior to the retrieval phase. Recognition memory performance was assessed using the discrimination index and exploratory preference. Immediately following behavioral testing, hippocampal tissue was collected for Western blot analysis of phosphorylated and total CaMKII-α, CaMKII-β, and ERK.

**Results:**

Scopolamine significantly impaired recognition memory retrieval, as evidenced by a reduced discrimination index and decreased preference for the novel object, without diminishing overall exploratory activity. At the molecular level, scopolamine-induced cholinergic inhibition selectively decreased hippocampal phosphorylation of both CaMKII-α and CaMKII-β while increasing ERK phosphorylation, with no significant changes in total protein expression of any kinase.

**Conclusions:**

These findings identify a retrieval-specific, scopolamine-induced molecular signature of cholinergic inhibition in the hippocampus, characterized by concurrent suppression of both CaMKII isoform activities (α and β) alongside dissociable ERK hyperactivation. By extending prior molecular observations from aversively motivated paradigms to an ethologically relevant model of recognition memory, this study provides mechanistic insight into how cholinergic dysfunction contributes to memory retrieval deficits and suggests that cholinergic signaling maintains the functional (CaMKII-α) and structural (CaMKII-β) dimensions of hippocampal memory retrieval machinery through coordinated kinase activation.

**Graphical Abstract:**

**Figure:**
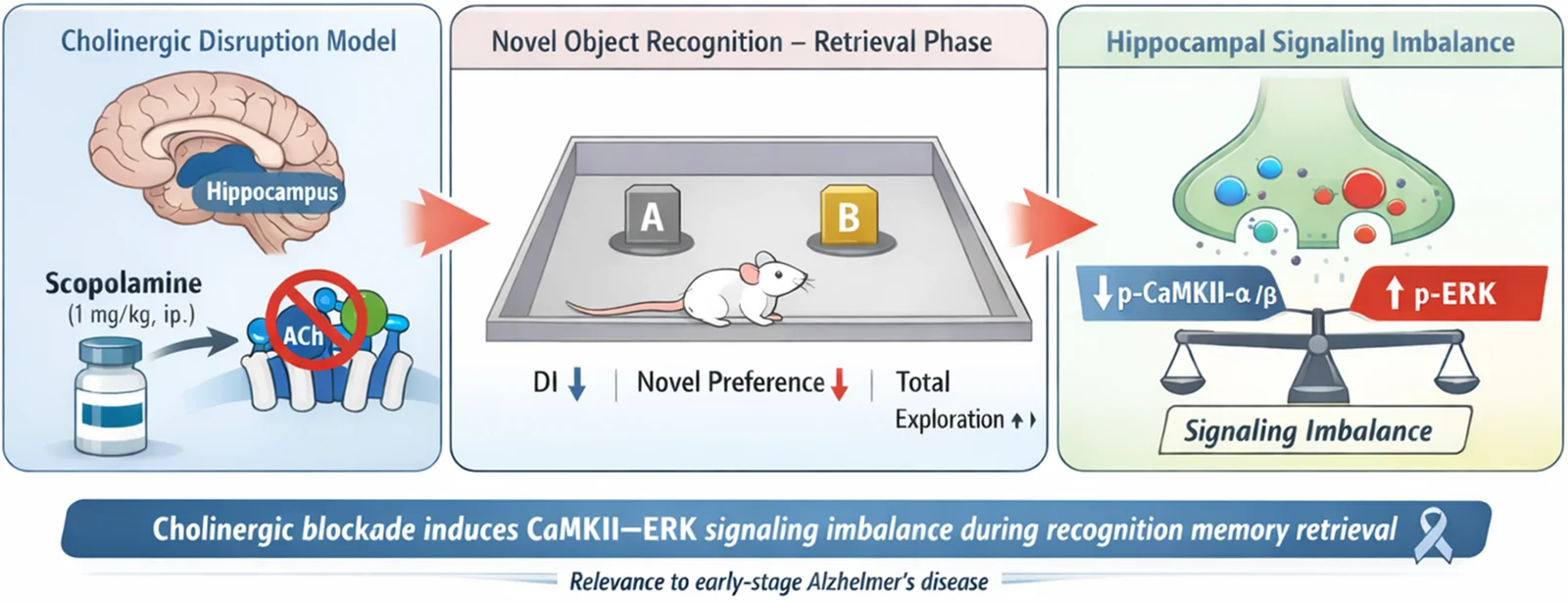

**Supplementary Information:**

The online version contains supplementary material available at https://doi.org/10.1186/s12868-026-01027-0.

## Introduction

Alzheimer’s disease (AD) is the leading cause of dementia worldwide and is characterized by progressive impairments in memory, learning, and executive function, with deficits in episodic and recognition memory emerging early in the disease course [1]. A prominent and long-standing hypothesis in AD research posits that degeneration of the basal forebrain cholinergic system and subsequent disruption of acetylcholine-mediated neurotransmission represent a major contributor to cognitive decline [2].

Although cholinergic signaling has been extensively studied in the context of memory encoding and consolidation, its specific contribution to memory retrieval remains comparatively underexplored. This gap is particularly relevant given that retrieval failure—the inability to access previously stored information—is a hallmark feature of cognitive impairment in AD and related dementias [3]. Importantly, retrieval deficits in AD can occur even when encoding and consolidation remain relatively intact, highlighting the need to understand phase-specific mechanisms [4]. Notably, pharmacological enhancement of cholinergic transmission via acetylcholinesterase inhibitors has been shown to improve retrieval-related memory performance in patients with AD, further implicating cholinergic mechanisms in this phase of memory processing [5, 6].

Scopolamine is a non-selective competitive antagonist at all five muscarinic acetylcholine receptor subtypes (M1–M5) that readily crosses the blood-brain barrier [7]. At the dose used in the present study (1 mg/kg, i.p.), scopolamine produces transient (~ 4–6 h) blockade of central muscarinic receptors, with peak brain concentrations occurring approximately 30 min after systemic administration [7]. By inhibiting M1 receptor-mediated signaling—which is critically involved in hippocampal-dependent cognitive processes—scopolamine provides a well-established pharmacological approach for investigating the role of cholinergic transmission in memory function [7, 8]. While scopolamine-induced cognitive impairment is not considered a model of AD per se, it allows dissection of the molecular consequences of acute cholinergic hypofunction that are relevant to the retrieval deficits observed in conditions characterized by cholinergic degeneration, including AD [9]. Pharmacological antagonism of muscarinic acetylcholine receptors using scopolamine is widely employed to investigate the role of cholinergic signaling in cognitive processes and reliably induces memory impairments across a range of behavioral paradigms [9]. However, findings regarding the impact of muscarinic blockade on memory retrieval have been mixed, with reports of minimal effects in some contexts [10, 11] and robust impairments in others, including spatial learning, fear conditioning, and passive avoidance tasks [12–15]. These paradigms often rely on conditioned or emotionally salient associations, which may engage neural and molecular mechanisms distinct from those underlying spontaneous recognition memories.

The novel object recognition (NOR) task provides an ethologically relevant, non-aversive measure of recognition memory that capitalizes on rodents’ innate preference for novelty and engages neural circuits involving both the hippocampus and perirhinal cortex [16, 17]. Although the perirhinal cortex plays a prominent role in object familiarity, hippocampal signaling is critically engaged during retrieval under conditions requiring contextual integration and novelty discrimination. While cholinergic modulation of NOR performance has been primarily investigated during encoding and consolidation, relatively few studies have isolated its effects during the retrieval phase. Notably, scopolamine administration prior to testing has been shown to disrupt NOR performance [18], yet the molecular mechanisms underlying this effect remain poorly characterized.

At the molecular level, calcium/calmodulin-dependent protein kinase II alpha (CaMKII-α) and extracellular signal-regulated kinase (ERK) represent key signaling nodes that integrate synaptic activity and regulate long-term synaptic plasticity [19, 20]. Activation of CaMKII-α, driven by calcium influx through NMDA receptors, is essential for the induction and maintenance of long-term potentiation (LTP) and the stabilization of memory-related synaptic ensembles [20]. ERK signaling, in parallel, contributes to activity-dependent transcriptional and structural remodeling associated with memory formation and consolidation, although dysregulated or temporally inappropriate ERK activation has been implicated in cognitive dysfunction and neurodegenerative pathology [19].

In addition to CaMKII-α, the hippocampus expresses abundant levels of the closely related CaMKII-β isoform. While CaMKII-α is primarily enriched at the postsynaptic density and directly phosphorylates synaptic targets such as AMPA receptors to enhance synaptic strength, CaMKII-β exhibits high-affinity binding to F-actin and plays a distinct role in regulating dendritic spine morphology and stability [21, 22]. Phosphorylation of CaMKII-β at its autophosphorylation site (Thr287) is required for its activity-dependent translocation to synapses and for the structural plasticity underlying long-term memory maintenance [23]. Notably, dysregulation of CaMKII-β signaling has been implicated in spine loss and cognitive decline in models of neurodegeneration [24, 25], further underscoring the potential relevance of this isoform to conditions involving cholinergic dysfunction. Despite these complementary functions, nearly all studies of cholinergic-dependent memory retrieval have focused exclusively on CaMKII-α [26], leaving the regulation of CaMKII-β under conditions of acute cholinergic blockade entirely unexplored.

Previous work in aversively motivated paradigms, particularly the passive avoidance task, has demonstrated that cholinergic blockade during retrieval is associated with alterations in hippocampal CaMKII-α and ERK signaling [14, 15, 27]. However, whether a comparable, retrieval-phase–specific molecular signature underlies recognition memory deficits in the NOR task—a paradigm that minimizes stress and motivational confounds—remains unknown. To date, no study has directly examined the phase-specific regulation of hippocampal CaMKII-α and ERK signaling during NOR retrieval under conditions of cholinergic inhibition. Moreover, the potential involvement of CaMKII-β —a key regulator of spine architecture— has not been investigated in any retrieval paradigm following cholinergic blockade via scopolamine treatment.

Accordingly, the present study aimed to determine whether acute scopolamine administration impairs retrieval in the NOR task and to characterize the associated changes in hippocampal CaMKII-α, CaMKII-β, and ERK phosphorylation. We hypothesized that scopolamine-induced cholinergic inhibition would disrupt recognition memory retrieval and induce a dissociable pattern of kinase regulation, reflected by reduced activation of both CaMKII isoforms (α and β) and enhanced ERK phosphorylation in the hippocampus.

## Materials and methods

### Chemicals

Scopolamine hydrochloride (S-1013) was purchased from Sigma Aldrich, USA. Western blot antibodies including phosphorylated-CaMKII (p-CaMKII, #12716, detects both α and β isoforms), CaMKII (pan) (#4436, detects both α (~ 50 kDa) and β (~ 60 kDa) isoforms), phosphorylated-ERK (p-ERK #4377), ERK (#4695), and secondary HRP-conjugated (#7074) antibodies were prepared by Cell Signaling Technology Company (Denver, MA, USA). GAPDH (Glyceraldehyde 3-phosphate dehydrogenase) antibody (mouse mAb, #sc-166574) was from Santa Cruz Biotechnology. Anti-Mouse HRP-linked Antibody (#S554002) was from BD Pharmingen™ company. Amersham ECL select reagent kit (RPN2235) was purchased from GE Healthcare Life Sciences, UK. Halt protease and phosphatase inhibitor cocktail was from Thermo Scientific (78445) and PVDF membrane (IPVH00010) was purchased from Millipore. Other reagents were obtained from usual commercial sources.

### Animals

Adult male SWR/J mice (25–30 g, 8–10 weeks of age upon arrival) were sourced from the animal facility of Shiraz University of Medical Sciences, Shiraz, Iran. Mice were allowed to acclimate to the housing facility for one week prior to experimental procedures, and were 9–11 weeks of age at the time of behavioral testing. To minimize potential variability associated with estrous cycle-dependent hormonal fluctuations, which have been shown to influence memory performance in female rodents [28], only male mice were used in this initial mechanistic study. The animals had access to water and food *ad libitum*, and were housed under controlled temperature (20 ± 2 °C) and lighting (07:00 to 19:00 h) status. All procedures in this study were conducted in accordance with the Research Ethics Committee of Shiraz University of Medical Sciences, Shiraz, Iran (license # IR.SUMS.AEC.1400.023 and IR.SUMS.AEC.1402.024). All animal experiments were carried out in accordance with National Institutes of Health guide for the care and use of Laboratory animals (NIH Publications 8th edition, 2011).

### Experimental design and drug administration

**Fig. 1:**
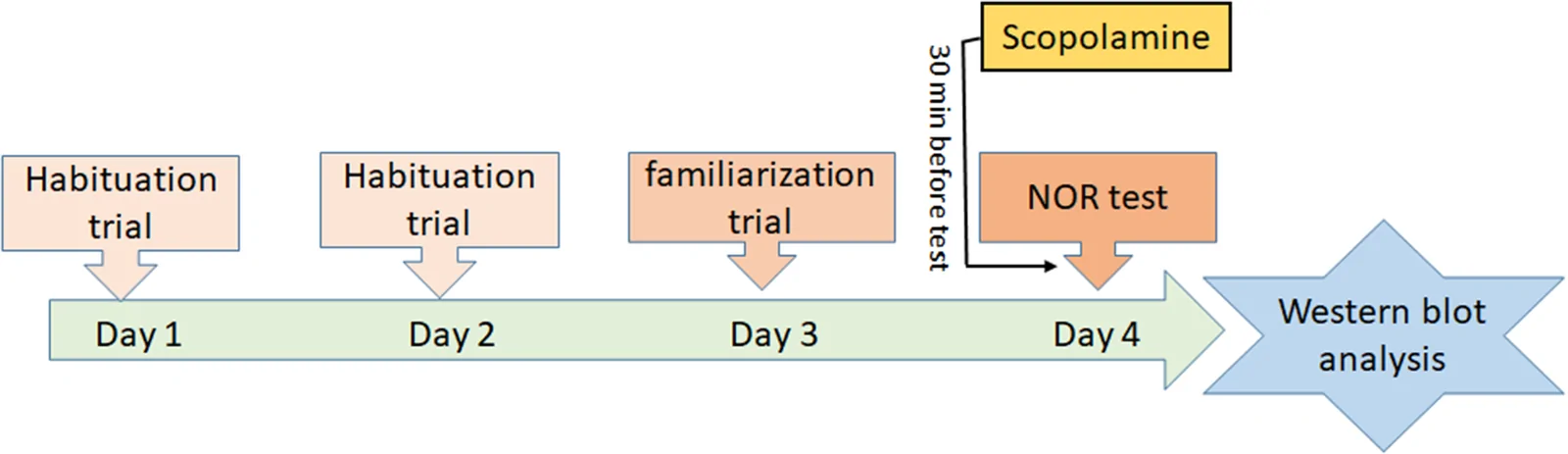
Experimental timeline and study design. Schematic representation of the experimental protocol illustrating the timeline of habituation, familiarization, and test phases of the novel object recognition (NOR) task, along with the timing of scopolamine or saline administration and subsequent hippocampal tissue collection for biochemical analyses. Scopolamine (1 mg/kg, i.p.) or vehicle was administered 30 min prior to the NOR test phase, followed by euthanasia and hippocampal dissection for Western blot assessment of CaMKII-α, CaMKII-β, and ERK signaling pathways

The animals were randomly assigned into 2 groups (*n* = 8/group) and the experimenters were blind to the treatment conditions (Fig. 1). Scopolamine hydrochloride was dissolved in sterile saline and administered intraperitoneally (i.p.) at a dose of 1 mg/kg (injection volume: 10 mL/kg body weight). The control group received an equivalent volume of sterile saline. Both injections were given 30 min prior to the NOR test phase.

### Novel object recognition (NOR) task

The NOR task is widely used to evaluate recognition memory in mice [17, 29]. The NOR apparatus consisted of a 40 × 40 cm open field arena custom-built from Plexiglas, was employed over 4 days, starting with 2 days of habituation sessions where mice were acclimatized to the field. Day 3 involved a familiarization trial in which mice interacted with two identical 4 cm high plastic objects (A1 and A2) for 10 min [30]. Exploration was defined as sniffing or touching the object with the nose or forepaws; climbing on the object was not counted as exploration. Mice that did not reach an exploration time of at least 20 s were excluded from the study [17]. The NOR test was performed 24 h later, during which one of the familiar objects (object A2) was replaced by a novel object (object B, the same size as the familiar object, with a different shape). The test session lasted 10 min. The objects and their locations in the apparatus were counterbalanced to ensure the same use as the novel or familiar objects. After each experiment, all the objects and the field were cleaned with 70% ethanol. Discrimination index (DI) was calculated as (Tnovel − Tfamiliar) / (Tnovel + Tfamiliar), where Tnovel and Tfamiliar represent the time spent exploring the novel and familiar objects, respectively. Exploratory preference was calculated as [Tnovel / (Tnovel + Tfamiliar)] × 100, representing the percentage of total exploration time directed toward the novel object.

### Tissue preparation

Immediately following the behavioral tests, the mice were euthanized using carbon dioxide inhalation. The brain was dissected into respective hemispheres and the cortex area was retracted to isolate the hippocampus. Hippocampi were dissected on ice, snap frozen in liquid nitrogen and stored at -80 °C. Behavioral analyses were performed in *n* = 8 mice/group. For Western blot analyses, due to the limited tissue yield from individual mouse hippocampi, 5–6 samples per group were used. This sample size is consistent with previous studies employing hippocampal Western blotting in mice [31, 32].

### Western blot analysis

Western blotting is a widely used technique in molecular biology to detect proteins in biological tissues [33]. Hippocampal tissues were homogenized and lysed in cold RIPA lysis buffer containing protease and phosphatase inhibitors. The tissue lysate was then centrifuged (12,000 rpm, 20 min., 4 °C), and the amount of protein in the supernatant was measured using the modified Lowry method [34, 35]. The western blot procedure followed previous protocols [36, 37]. Protein samples (50 µg for each sample) were electrophoresed on 10% sodium dodecyl sulfate polyacrylamide gel electrophoresis (SDS-PAGE) to separate protein macromolecules and fragments, based on size and charge [38]. Samples were then transferred to polyvinylidene difluoride (PVDF) membranes using a rapid protein transfer apparatus (Trans-Blot Turbo Transfer System, Bio-Rad). To prevent nonspecific binding of antibodies to the membrane, each membrane was blocked (1 h, RT) with bovine serum albumin (BSA) at 3% in a buffer mixture (Tris -buffered saline and polysorbate 20 (TBST)). Then each membrane was incubated in primary antibodies: p-ERK (1/2000), ERK (1/3000), p-CaMKII (1/2000, detects both α and β isoforms), CaMKII (1/3000, detects both α and β isoforms), and GAPDH (1/2000) overnight at 4 °C. After washing the membranes with TBST, the blots were incubated in corresponding HRP-conjugated secondary antibodies for 2 h at room temperature. Protein bands were visualized using enhanced chemiluminescence (ECL) reagents. Given that the CaMKII antibody recognizes both α (~ 50 kDa) and β (~ 60 kDa) isoforms, bands corresponding to each isoform were quantified separately using ImageJ software [39, 40]. Phosphorylated and total protein levels for each isoform were normalized to GAPDH, and the ratio of phosphorylated to total protein was calculated as an index of activation. The mean level of the control group was set to 1 as a baseline, and the other experimental conditions were compared to this standard.

### Data analysis

Data were analyzed using GraphPad Prism 9. Normality was assessed using the Shapiro–Wilk test, and homogeneity of variances was evaluated using Levene’s test. For variables that met the assumptions of normality (total exploration time and all Western blot data), between-group comparisons were performed using unpaired two-tailed Student’s t-tests. For variables that showed significant deviations from normality (discrimination index and exploratory preference in both familiarization and test phases), the non-parametric Mann–Whitney U test was used. *P* < 0.05 was considered statistically significant.

## Results

### Effect of scopolamine on retrieval performance in the NOR task

**Fig. 2:**
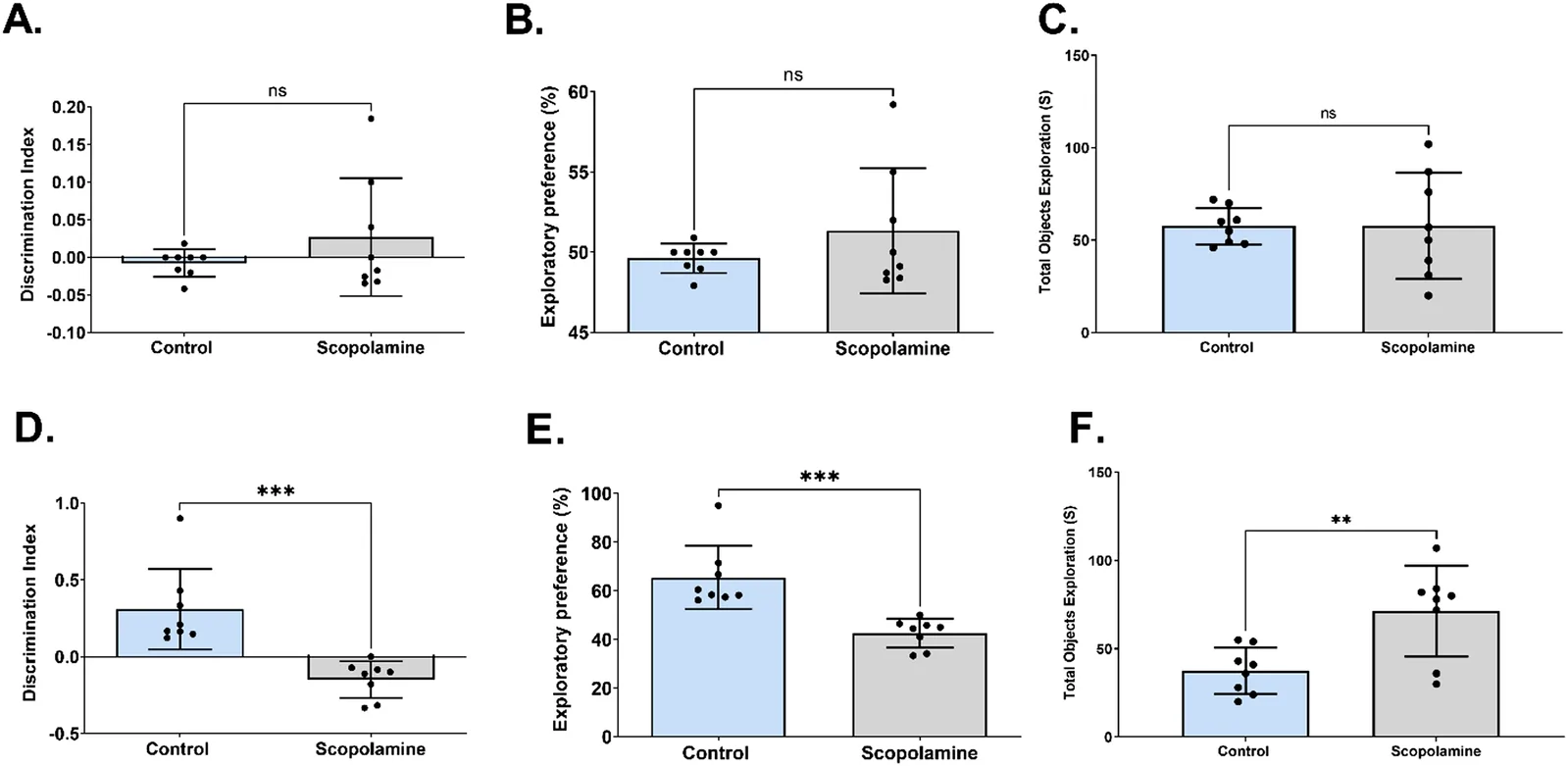
Effects of scopolamine on retrieval performance in the novel object recognition task. (**A**) Discrimination index (DI), (**B**) exploratory preference for object A1, and (**C**) total exploration time during the familiarization (acquisition) phase, showing no significant baseline differences between groups. (**D**) DI and (**E**) exploratory preference for novel object during the test phase, demonstrating a significant impairment in recognition memory following scopolamine administration. (**F**) Total exploration time during the test phase, indicating increased exploratory activity in scopolamine-treated mice relative to controls. Discrimination index and exploratory preference were analyzed using the Mann–Whitney U test; total exploration time was analyzed using unpaired Student's t-test. Data are presented as mean ± SD (n = 8 per group). ** p < 0.01, ***p < 0.001 versus control

As shown in Fig. 2, no significant differences were observed between groups in the discrimination index (Mann–Whitney U = 30, *p* = 0.8766), exploratory preference (Mann–Whitney U = 30, *p* = 0.8766; 49.6% vs. 51.3%), or total exploration time (t(14) = 0.1171, *p* > 0.05) during the familiarization phase (Figs. 2A–C). Because the discrimination index and exploratory preference are derived from the same raw exploration times, their Mann–Whitney U statistics are identical. In contrast, administration of scopolamine 30 min prior to the test phase resulted in a marked impairment in recognition memory.

Specifically, scopolamine-treated mice exhibited a significant reduction in the DI compared with controls (Fig. 2D; Mann–Whitney U = 0, *p* = 0.0002), accompanied by a significant decrease in exploratory preference for the novel object (Mann–Whitney U = 0, *p* = 0.0002; 65.5% in controls vs. 42.5% in scopolamine-treated mice; Fig. 2E).

To exclude the possibility that these deficits reflected altered general motivation or locomotor activity, total exploration time during the test phase was analyzed (Fig. 2F). Scopolamine treatment significantly increased total exploration time relative to controls (*t* (14) = 3.285, *p* < 0.01), indicating that impaired performance in the NOR task was not attributable to reduced exploratory drive. Instead, these findings suggest a selective deficit in the ability to discriminate between familiar and novel objects, confirming that acute cholinergic blockade impairs recognition memory retrieval in the NOR task.

Importantly, no significant differences in total exploration time were observed between groups during the familiarization phase (Fig. 2C), confirming comparable baseline exploratory behavior prior to pharmacological intervention.

### Scopolamine reduces phosphorylated CaMKII-α and CaMKII-β levels in the hippocampus

**Fig. 3:**
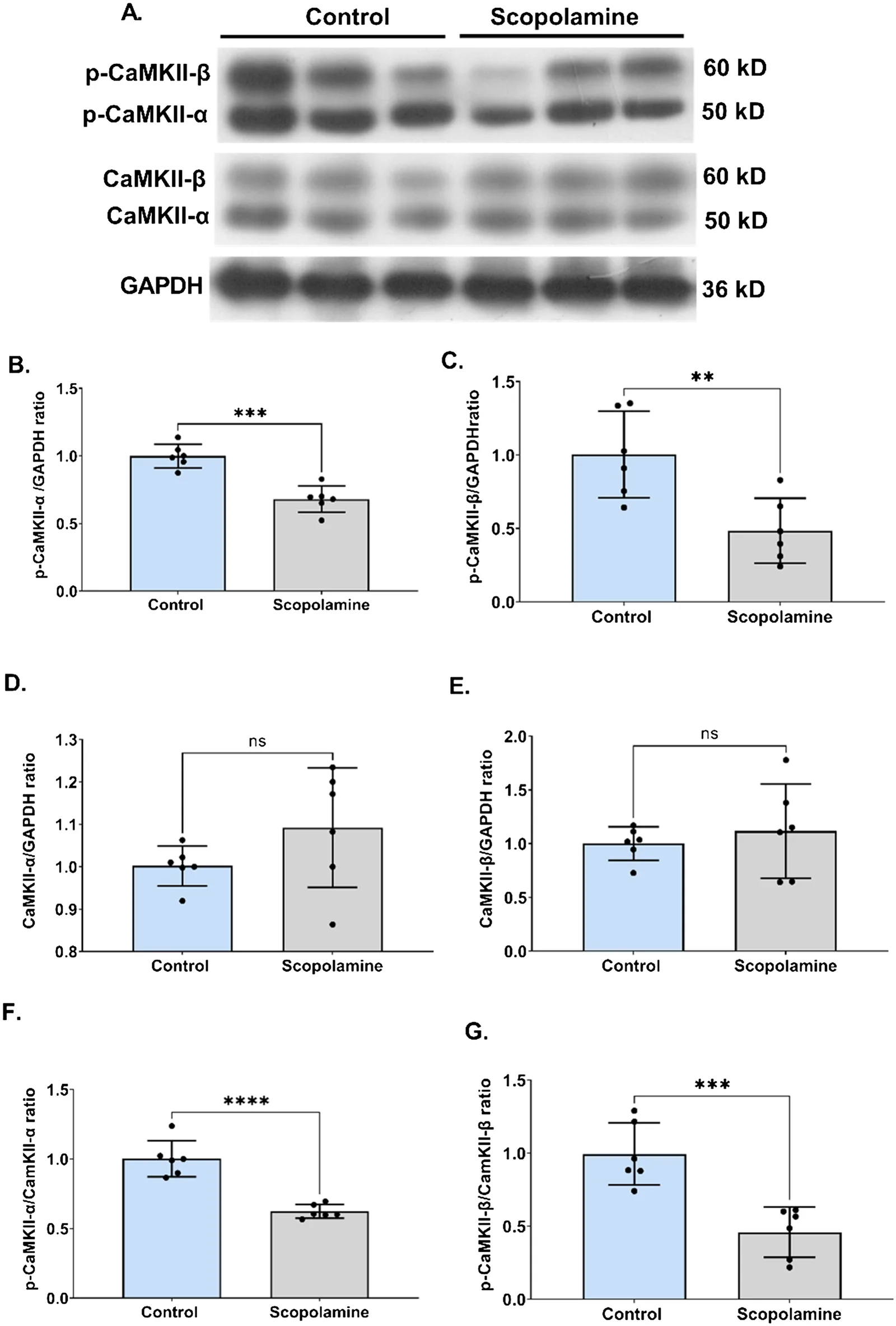
Effects of scopolamine on hippocampal CaMKII-α and CaMKII-β signaling. (**A**) Representative Western blot images showing phosphorylated CaMKII (p-CaMKII) and total CaMKII for both α (~ 50 kDa) and β (~ 60 kDa) isoforms, along with GAPDH loading control, in hippocampal lysates from control and scopolamine-treated mice. **(B**) Quantification of p-CaMKII-α levels normalized to GAPDH. (**C**) Quantification of p-CaMKII-β levels normalized to GAPDH. (**D**) Quantification of total CaMKII-α levels normalized to GAPDH. (**E**) Quantification of total CaMKII-β levels normalized to GAPDH. (F) Ratio of p-CaMKII-α to total CaMKII-α, reflecting CaMKII-α activation status. (**G**) Ratio of p-CaMKII-β to total CaMKII-β, reflecting CaMKII-β activation status. Data are expressed as fold change relative to the control group (set to 1.0) and presented as mean ± SD (*n* = 6 per group). Statistical analysis was conducted using unpaired Student’s t-tests. **p < 0.01, ***p < 0.001, ****p < 0.0001 versus control. Uncropped blots are provided in the Supplementary Material

Western blot analysis revealed distinct bands corresponding to phosphorylated CaMKII (p-CaMKII) and total CaMKII for both the α (~ 50 kDa) and β (~ 60 kDa) isoforms in hippocampal lysates (Fig. 3A). Pre-test administration of scopolamine significantly reduced hippocampal p-CaMKII-α levels relative to controls (Fig. 3B; t(10) = 5.954, *p* = 0.0001, Cohen’s d = 3.44). Similarly, p-CaMKII-β levels were also significantly decreased in scopolamine-treated mice (Fig. 3C; t(10) = 3.452, *p* = 0.0062, Cohen’s d = 1.99).

In contrast, total CaMKII-α (Fig. 3D) and total CaMKII-β (Fig. 3E) protein levels did not differ significantly between groups, indicating that scopolamine selectively attenuates the activation state of both CaMKII isoforms without altering their total expression levels.

Consistent with these findings, the ratios of p-CaMKII-α to total CaMKII-α (Fig. 3F; t(10) = 6.667, *p* < 0.0001, Cohen’s d = 3.85) and p-CaMKII-β to total CaMKII-β (Fig. 3G; t(10) = 4.788, *p* = 0.0007, Cohen’s d = 2.76), used as indices of kinase activation, were both significantly decreased in the scopolamine-treated group.

These results indicate that scopolamine-induced cholinergic blockade selectively attenuates both CaMKII-α and CaMKII-β activation in the hippocampus without altering total protein expression for either isoform. Uncropped Western blot images are provided in the Supplementary Material.

### Scopolamine increases ERK phosphorylation in the hippocampus

**Fig. 4:**
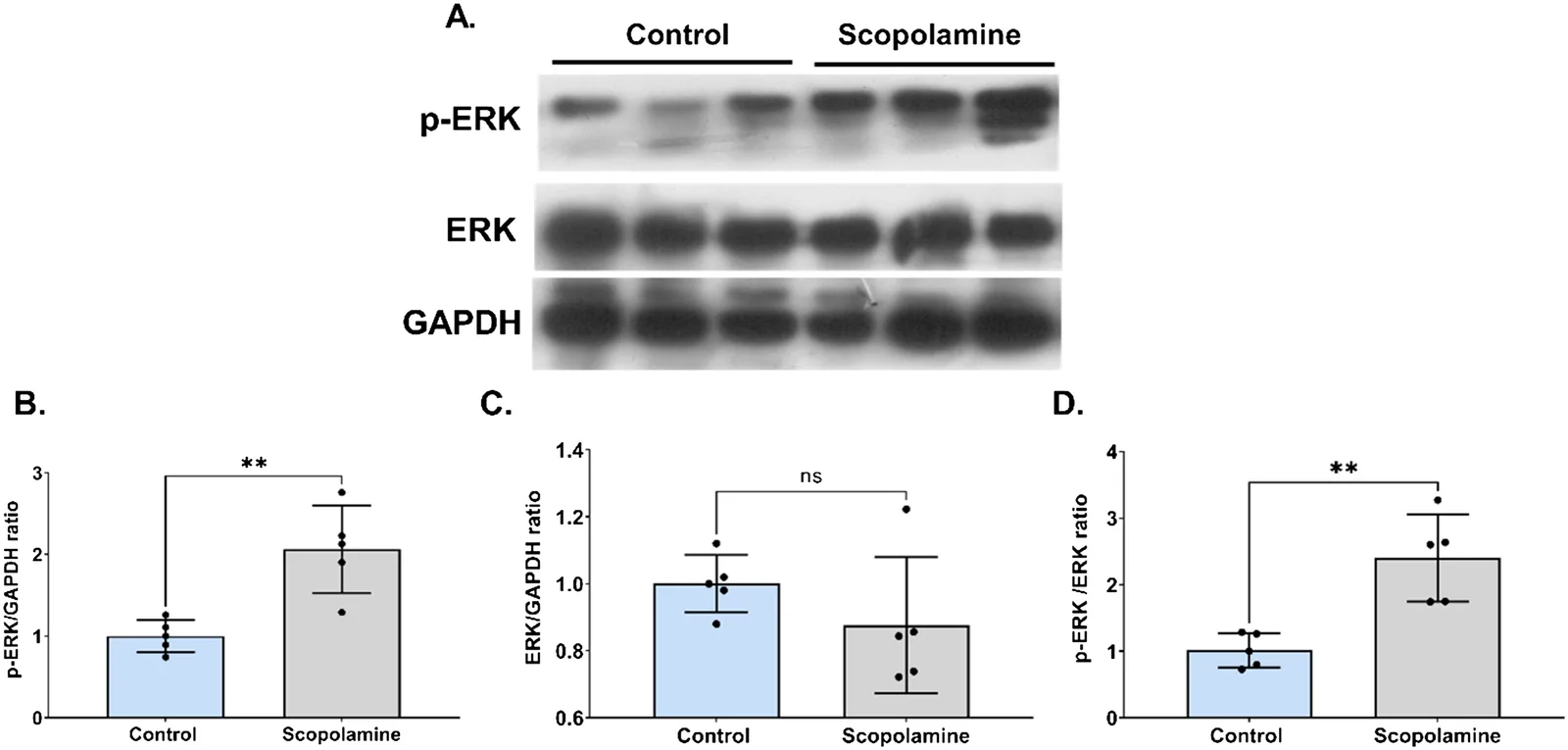
Effects of scopolamine on hippocampal ERK signaling. (**A**) Representative Western blot images of phosphorylated ERK (p-ERK), total ERK, and GAPDH in hippocampal tissue from control and scopolamine-treated mice. (**B**) Densitometric analysis of p-ERK normalized to GAPDH. (**C**) Quantification of total ERK normalized to GAPDH. (**D**) Ratio of p-ERK to total ERK, indicating ERK pathway activation. Data are shown as fold change relative to the control group and presented as mean ± SD (*n* = 5 per group). Statistical significance was determined using unpaired Student’s *t*-tests. ** *p* < 0.01 versus control. Full-length blots are included in the Supplementary Material

Representative immunoblots of phosphorylated ERK (p-ERK), total ERK, and GAPDH are shown in Fig. 4A. Quantitative analysis demonstrated a significant increase in hippocampal p-ERK levels following scopolamine administration compared with controls (Fig. 4B; t(8) = 4.172, *p* = 0.0031, Cohen’s d = 2.64).

While total ERK expression remained unchanged between groups (Fig. 4C), the ratio of p-ERK to total ERK was significantly elevated in the scopolamine-treated mice (Fig. 4D; t(8) = 4.408, *p* = 0.0023, Cohen’s d = 2.79), indicating enhanced ERK pathway activation in response to scopolamine-induced cholinergic inhibition. Uncropped Western blot images are available in the Supplementary Material.

## Discussion

The present study demonstrates that acute cholinergic blockade via scopolamine selectively impairs recognition memory retrieval, and this behavioral deficit is accompanied by a distinct pattern of hippocampal kinase dysregulation, characterized by reduced phosphorylation of both CaMKII-α and CaMKII-β together with increased ERK activation.

Scopolamine-induced impairments in memory performance have been extensively documented in aversively motivated paradigms, particularly the passive avoidance task, where cholinergic inhibition disrupts retrieval and is accompanied by alterations in hippocampal kinase signaling, including both CaMKII-α and ERK pathways [14, 15, 27]. However, despite the widespread use of the NOR task as a translational model of recognition memory, the molecular correlates of scopolamine-induced retrieval deficits in this paradigm have remained largely unexplored. The present findings bridge this gap by demonstrating that cholinergic inhibition elicits a comparable, yet contextually distinct, molecular signature in the hippocampus during the retrieval phase of NOR memory.

Cholinergic signaling plays a well-established role in hippocampal-dependent memory processes, and its disruption has been strongly implicated in deficits of episodic and recognition memory [41]. Although acetylcholine has been widely studied in the context of memory acquisition and consolidation, its role in retrieval-related processes is comparatively less defined. Evidence from experimental models indicates that direct optogenetic activation of hippocampal memory engram cells can restore memory recall even in the presence of severe encoding deficits [42], underscoring the importance of retrieval-specific molecular and network mechanisms. In parallel, hippocampal acetylcholine release has been shown to increase during novelty exploration in recognition memory paradigms [8], supporting a direct role for muscarinic signaling in modulating retrieval-related hippocampal activity.

At the molecular level, scopolamine-induced retrieval impairment in the NOR task was associated with a marked reduction in CaMKII-α phosphorylation without changes in total protein levels. Previous studies in passive avoidance paradigms have reported suppression of hippocampal CaMKII-α activity during retrieval following cholinergic blockade [43, 44]; the present data demonstrate that this mechanism generalizes to recognition memory retrieval in a non-aversive, exploratory-based task. This convergence suggests that CaMKII-α–dependent synaptic reactivation represents a shared molecular substrate for memory retrieval across distinct motivational and behavioral contexts.

Beyond the reduction in CaMKII-α phosphorylation, scopolamine treatment significantly decreased the phosphorylation state of CaMKII-β, as reflected by reduced p-CaMKII-β levels and a decreased p-CaMKII-β/total CaMKII-β ratio, with no change in total CaMKII-β protein expression. This finding is mechanistically distinct from a simple loss of protein and carries important functional implications. Phosphorylation of CaMKII-β at Thr287 is required for its translocation to synapses and for its ability to promote spine growth and stability [23]. Thus, reduced CaMKII-β phosphorylation following cholinergic blockade may impair the activity-dependent targeting of this isoform to dendritic spines, compromising the structural plasticity required for successful memory retrieval [45].

Notably, while total CaMKII-β protein remained unchanged in the present study—in contrast to some chronic neurodegeneration models where CaMKII-β expression is reduced—the selective reduction in its phosphorylation suggests that acute cholinergic blockade primarily affects the activation state rather than the stability of this isoform. This distinction is consistent with the rapid time course of scopolamine action (30 min), which is sufficient to alter kinase activity via upstream calcium-dependent mechanisms but likely insufficient to drive changes in protein turnover. Muscarinic M1 receptors tonically maintain intracellular calcium dynamics; their acute blockade may rapidly reduce calcium influx through NMDA receptors and voltage-gated calcium channels, thereby decreasing the calcium/calmodulin-dependent activation of both CaMKII isoforms [46].

Muscarinic acetylcholine receptors regulate intracellular calcium dynamics and modulate NMDA receptor function, both of which serve as critical upstream determinants of CaMKII-α activation [47]. Disruption of cholinergic signaling has been shown to perturb calcium homeostasis in hippocampal neurons [48, 49], providing a plausible mechanistic link between muscarinic receptor antagonism and reduced CaMKII-α phosphorylation. Of note, postmortem analyses of brain tissue from patients with AD have reported decreases in both CaMKII-α–expressing neurons and hippocampal CaMKII-α phosphorylation [24]. While these chronic neurodegenerative changes are distinct from the acute pharmacological manipulation employed here, the shared reduction in CaMKII-α activation raises the interesting—though speculative—question of whether cholinergic hypofunction contributes to CaMKII-α dysregulation across both acute and chronic conditions.

While both CaMKII isoforms were suppressed following cholinergic blockade, ERK signaling exhibited a distinct response pattern. In contrast to the suppression of CaMKII-α signaling, scopolamine treatment resulted in a significant increase in ERK phosphorylation in the hippocampus. Although ERK activation is widely associated with memory formation and consolidation, accumulating evidence indicates that its role is highly phase- and context-dependent. Hyperactivation of ERK has been reported in brain tissue from patients with AD and in experimental models of cognitive impairment [19, 50]. In passive avoidance paradigms, increased ERK signaling has been linked to retrieval deficits induced by cholinergic inhibition, and pharmacological suppression of ERK activity has been shown to ameliorate memory impairments in transgenic AD models [15, 51]. The present findings extend these observations to the NOR task, suggesting that dysregulated ERK signaling represents a convergent molecular feature of cholinergic-related retrieval failure across both aversive and non-aversive memory paradigms.

The concurrent decrease in CaMKII-α and CaMKII-β phosphorylation together with increased ERK phosphorylation observed in this study points to a decoupling of multiple plasticity-related signaling pathways under conditions of cholinergic inhibition. By simultaneously impairing both the functional potentiation (via CaMKII-α) and the structural support (via CaMKII-β) of memory-related synapses, while also driving aberrant ERK activation, this multi-pathway imbalance may disrupt the molecular fidelity required for successful discrimination between familiar and novel stimuli. While dysregulation of CaMKII-β phosphorylation has been independently linked to tau pathology and spine loss in models of neurodegeneration [25], whether the acute reduction in CaMKII-β phosphorylation observed here engages similar downstream mechanisms remains to be determined.

Several limitations should be considered. The use of a single scopolamine dose and a single post-retrieval time point precludes assessment of dose-dependent or temporal dynamics of kinase signaling. Moreover, while the observed associations between altered CaMKII-α/β and ERK activity and retrieval deficits are robust and statistically significant, the present study provides correlational evidence and cannot establish direct causal relationships. Definitive demonstration of causality would require pathway-specific pharmacological interventions (e.g., selective kinase inhibitors or activators), genetic manipulations (e.g., conditional knockout or overexpression), or site-specific manipulations of muscarinic receptor subtypes. Additionally, while we observed reduced phosphorylation of both CaMKII isoforms, the present study did not examine the subcellular localization of these isoforms; future studies using subcellular fractionation or immunohistochemistry will be necessary to determine whether cholinergic blockade differentially affects synaptic versus extrasynaptic pools of CaMKII-α and CaMKII-β. Future studies employing muscarinic receptor subtype–selective ligands, kinase inhibitors, or region-specific manipulations will be essential to directly test the functional contributions of these pathways to cholinergic-dependent recognition memory retrieval.

In conclusion, this study identifies a task-specific molecular signature of scopolamine-induced cholinergic inhibition in the hippocampus—characterized by suppressed phosphorylation of both CaMKII-α and CaMKII-β (with no change in total protein for either isoform) together with enhanced ERK phosphorylation—that accompanies selective impairment of retrieval in the NOR task. By extending prior molecular findings from aversively motivated paradigms to an ethologically relevant model of recognition memory, these results suggest that cholinergic signaling maintains memory retrieval through coordinated activation of both CaMKII isoforms, which are implicated in distinct aspects of synaptic plasticity—CaMKII-α in functional potentiation and CaMKII-β in structural reorganization [21, 23]. This framework highlights a conserved yet context-sensitive role for cholinergic modulation of intracellular signaling in memory retrieval and provides mechanistic insight into how cholinergic dysfunction may contribute to cognitive impairment.

## Supplementary Information

Below is the link to the electronic supplementary material.

Supplementary Material 1

## Data Availability

The datasets generated and/or analyzed during the current study are available from the corresponding author on reasonable request.
