# Supplementary material for: Cholinergic inhibition disrupts retrieval-specific phosphorylation of CaMKII-α and CaMKII-β alongside ERK hyperactivation in the hippocampus during novel object recognition memory
Source: BMC Neurosci. 2026 Jul 20;27:44. doi: 10.1186/s12868-026-01027-0 (PMC13670601; doi:10.1186/s12868-026-01027-0)
Supplement: Supplementary file 1 [file 12868_2026_1027_MOESM1_ESM.pdf]

Supplementary Material: Raw and Uncropped Western Blot Images

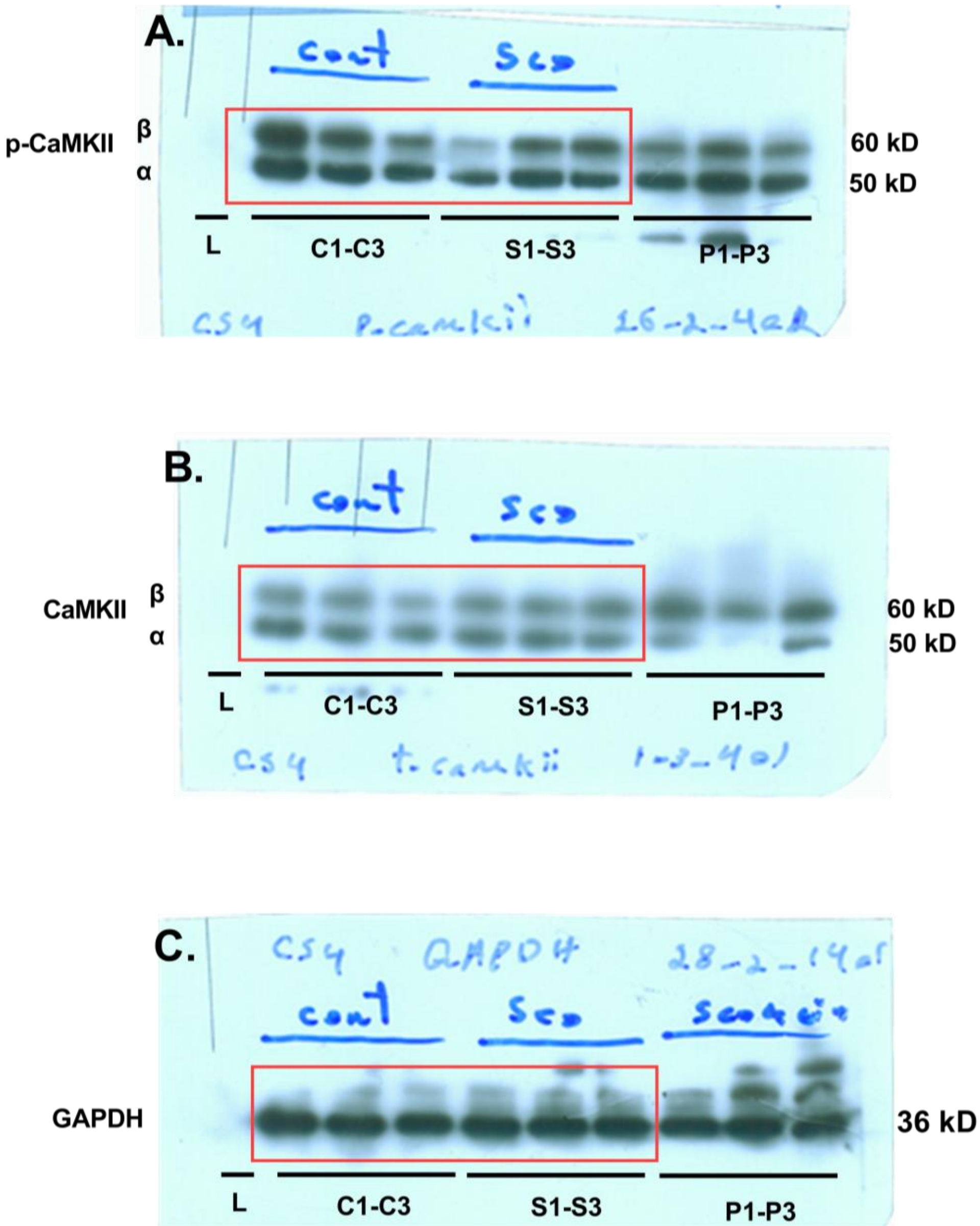

Figure S1. Uncropped Western blots for Figure 3A — CaMKII signaling.

(A) p-CaMKII (upper band: β, ~60 kDa; lower band: α, ~50 kDa), (B) total CaMKII (β and α), (C) GAPDH.

Lanes: L, molecular weight ladder; C1–C3, saline treated controls; S1–S3, scopolamine treated (1 mg/kg). Lanes P1–P3 contained samples from a separate pilot experiment not included in this study. The red box indicates the six lanes (C1–C3, S1–S3) shown in Figure 3A of the manuscript.

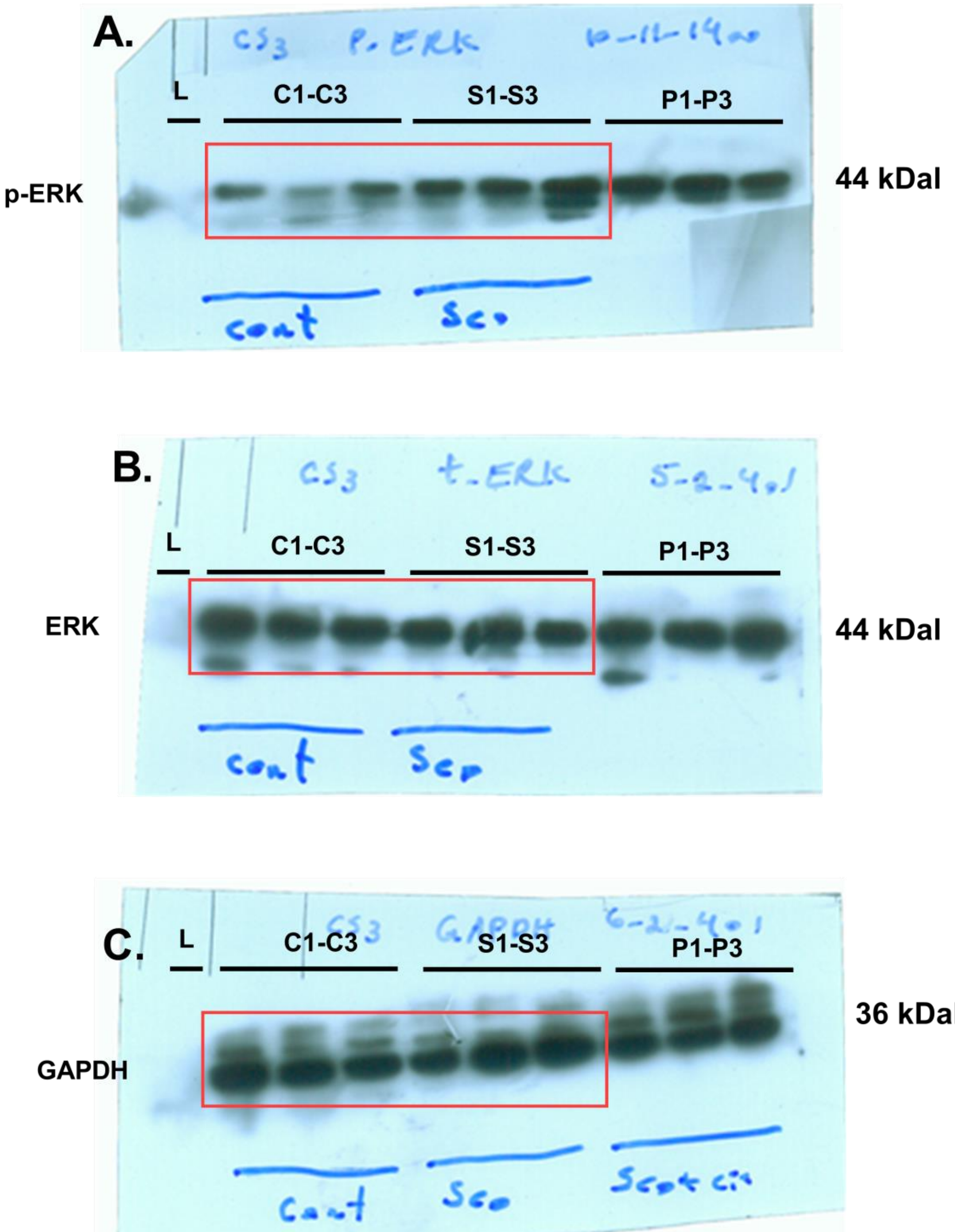

Figure S2. Uncropped Western blots for Figure 4A — ERK signaling.
(A) p-ERK, (B) total ERK, (C) GAPDH.
Lanes: L, molecular weight ladder; C1–C3, saline-treated controls; S1–S3, scopolamine-treated (1 mg/kg); P1–P3, samples from a separate pilot experiment not included in this study. The red box indicates the six lanes (C1–C3, S1–S3) shown in Figure 4A of the manuscript.
